# Crystal structure of bis­(azido-κ*N*)bis[2,5-bis(pyridin-2-yl)-1,3,4-thia­diazole-κ^2^ *N* ^2^,*N* ^3^]cobalt(II)

**DOI:** 10.1107/S2056989015006544

**Published:** 2015-04-09

**Authors:** Abdelhakim Laachir, Fouad Bentiss, Salaheddine Guesmi, Mohamed Saadi, Lahcen El Ammari

**Affiliations:** a Laboratoire de Chimie de Coordination et d’Analytique (LCCA), Faculté des Sciences, Université Chouaib Doukkali, BP 20, M-24000 El Jadida, Morocco; b Laboratoire de Catalyse et de Corrosion de Matériaux (LCCM), Faculté des Sciences, Université Chouaib Doukkali, BP 20, M-24000 El Jadida, Morocco; c Laboratoire de Chimie du Solide Appliquée, Faculté des Sciences, Université Mohammed V, Avenue Ibn Battouta, BP 1014, Rabat, Morocco

**Keywords:** crystal structure, transition metal, 2,5-bis­(pyridin-2-yl)-1,3,4-thia­diazole ligand, azide compounds, hydrogen bonding, π–π inter­actions

## Abstract

In the mononuclear title complex, [Co(N_3_)_2_(C_12_H_8_N_4_S)_2_], the cobalt(II) atom is located on an inversion centre and displays an axially weakly compressed octa­hedral coordination geometry. The equatorial positions are occupied by the N atoms of two 2,5-bis­(pyridin-2-yl)-1,3,4-thia­diazole ligands, whereas the axial positions are occupied by N atoms of the azide anions. The thia­diazole and pyridine rings linked to the metal are almost coplanar, with a maximum deviation from the mean plane of 0.0273 (16) Å. The cohesion of the crystal is ensured by weak C—H⋯N hydrogen bonds and by π–π inter­actions between pyridine rings [inter­centroid distance = 3.6356 (11) Å], forming a layered arrangement parallel to (001). The structure of the title compound is isotypic with that of the analogous nickel(II) complex [Laachir *et al.* (2013 ▸). *Acta Cryst.* E**69**, m351–m352].

## Abstract 2

The structure of the title compound is isotypic with that of the analogous nickel(II) complex, in which the CoN_6_ core shows an axially weakly compressed octa­hedral geometry as opposed to the almost regular geometry exhibited by the NiN_6_ octa­hedron.

## Chemical context   

In recent years, the use of the ligand 2,5-bis­(pyridin-2-yl)-1,3,4-thia­diazole has been studied for the synthesis of numerous complexes with transition-metal salts. An inter­esting feature of the metal–ligand chemistry of these compounds is that the resulting complexes can be mononuclear (Bentiss *et al.*, 2011*a* ▸; 2012 ▸; Kaase *et al.*, 2014 ▸) or binuclear (Bentiss *et al.*, 2004 ▸; Laachir *et al.*, 2013 ▸). Another preparation method involves the use of the organic ligand and pseudohalide ions, especially the azide ion which is known to exhibit different coordination modes (Nath & Baruah, 2012 ▸; Ray *et al.*, 2011 ▸).
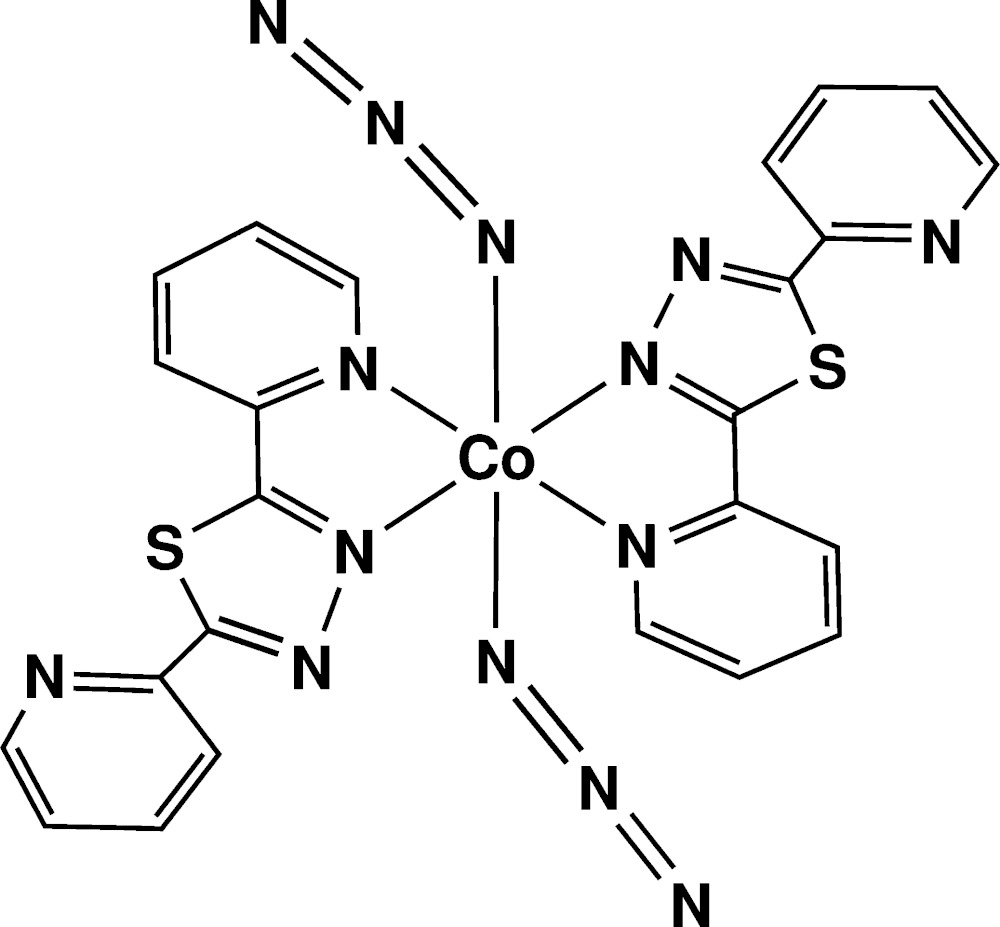


## Structural commentary   

**Figure 1:**
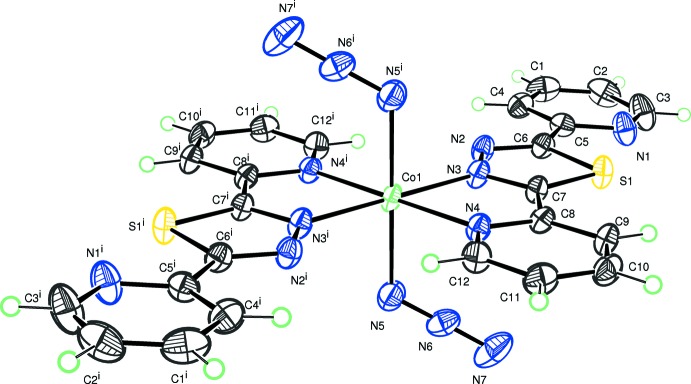
The mol­ecular structure of the title compound with displacement ellipsoids drawn at the 50% probability level. H atoms are represented as spheres of arbitrary radius. [Symmetry code: (i) −*x*, −*y*, −*z*.]

The structure of the title compound (Fig. 1 ▸) is isotypic with its nickel(II) analogue (Laachir *et al.*, 2015 ▸) and similar to that of the homologous compound, [Co(C_12_H_8_N_4_S)_2_(H_2_O)_2_]·2BF_4_, in which the water mol­ecules are substituted by azide ions which at the same time neutralize the positive charge of Co^2+^ (Bentiss *et al.*, 2011*b* ▸). The main difference between the two structures lies in the values of the dihedral angle between the two pyridine rings: this is 18.72 (6)° in the hydrated mol­ecule, whereas it is 3.03 (2)° in the title mol­ecule, (I). The dihedral angles formed by the thia­diazole ring and the pyridine rings N1/C1–C4 and N2/C8–C11 in (I) are 2.87 (9) and 1.1 (2)°, respectively. The cobalt cation, which is located on an inversion centre, shows an axially weakly compressed octa­hedral coordination geometry with the equatorial plane provided by four nitro­gen atoms belonging to the pyridine and thia­diazole rings of two organic ligands [Co1—N3 = 2.1301 (14) and Co1—N4 = 2.1535 (14) Å] and the axial positions occupied by two nitro­gen atoms from azide anions [Co1—N5 = 2.1132 (17) Å].

## Supra­molecular features   

**Figure 2:**
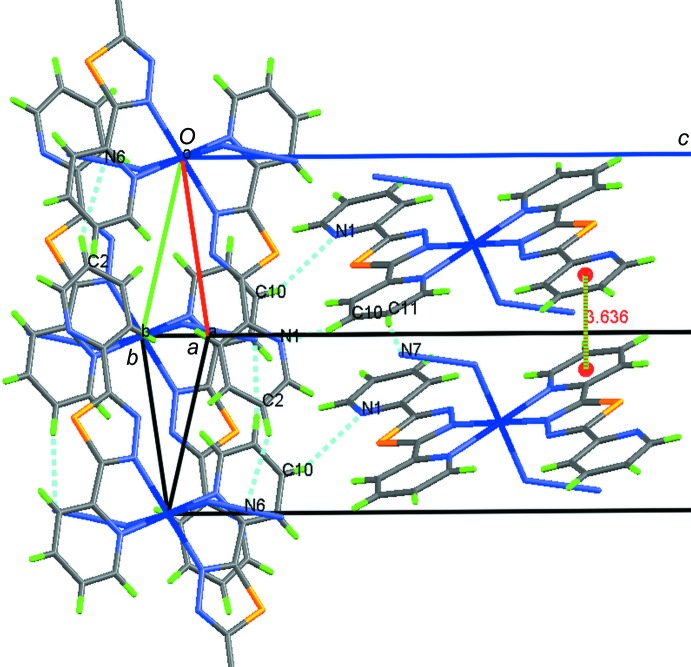
Partial crystal packing of the title compound, showing inter­molecular π–π inter­actions between pyridine rings (dashed green lines) and inter­molecular C—H⋯N hydrogen bonds (dashed blue lines).

In the crystal, the mol­ecules are linked by π–π inter­actions between pyridine rings [inter­centroid distance = 3.6356 (11) Å] and by weak C—H⋯N hydrogen bonds (Table 1 ▸), forming a layered arrangement parallel to (001) (Fig. 2 ▸). The layers are connected by further C—H⋯N hydrogen bonds into a three-dimensional network.

## Synthesis and crystallization   

The ligand 2,5-bis­(pyridin-2-yl)-1,3,4-thia­diazole (noted *L*) was synthesized as described previously by Lebrini *et al.* (2005 ▸). The complex [Co*L*_2_(N_3_)_2_] was synthesized in bulk qu­antity by dropwise addition with constant stirring at room temperature of an aqueous solution of NaN_3_ (0.4 mmol, 26 mg) to an ethanol/water solution (1:1 *v*/*v*) of *L* (0.1 mmol, 24 mg) and CoCl_2_·6H_2_O (0.1 mmol, 24 mg). The red-coloured solid precipitated was filtered and washed with cold ethanol. Single crystals of the title compound suitable for X-ray data collection were obtained by slow inter­diffusion of a solution of CoCl_2_·6H_2_O and *L* in aceto­nitrile into NaN_3_ dissolved in water. Red block-shaped single crystals appeared after one month. The crystals were washed with water and dried under vacuum (yield 60%). Analysis calculated for C_24_H_16_N_14_CoS_2_: C, 46.23; H, 2.59; N, 31.45 S, 10.28. Found: C, 46.42; H, 2.63; N, 31.35; S, 10.51.

**CAUTION!** Azide compounds are potentially explosive. Only a small amount of material should be prepared and handled with care.

## Refinement   

Crystal data, data collection and structure refinement details are summarized in Table 2 ▸. H atoms were located in a difference Fourier map and treated as riding, with C—H = 0.93 Å, and with *U*_iso_(H) = 1.2 *U*_eq_(C). Two outliers (002 and 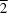
24) were omitted in the last cycles of refinement.

## Supplementary Material

Crystal structure: contains datablock(s) I. DOI: 10.1107/S2056989015006544/rz5153sup1.cif

Structure factors: contains datablock(s) I. DOI: 10.1107/S2056989015006544/rz5153Isup2.hkl

CCDC reference: 1057234

Additional supporting information:  crystallographic information; 3D view; checkCIF report

## Figures and Tables

**Table 1:** Hydrogen-bond geometry (, )

*D*H*A*	*D*H	H*A*	*D* *A*	*D*H*A*
C2H2N6^i^	0.93	2.59	3.432(3)	151
C11H11N7^ii^	0.93	2.60	3.528(3)	173
C10H10N1^iii^	0.93	2.63	3.438(2)	146

Symmetry codes: (i) 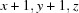; (ii) 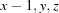; (iii) 
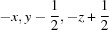
.

**Table 2:** Experimental details

Crystal data	
Chemical formula	[Co(N_3_)_2_(C_12_H_8_N_4_S)_2_]
*M*_r_	623.56
Crystal system, space group	Monoclinic, *P*2_1_/*c*
Temperature (K)	296
*a*, *b*, *c* ()	7.8004(3), 8.2439(3), 20.3222(8)
()	92.910(2)
*V* (^3^)	1305.15(9)
*Z*	2
Radiation type	Mo *K*
(mm^1^)	0.86
Crystal size (mm)	0.39 0.31 0.18

Data collection	
Diffractometer	Bruker APEXII CCD
Absorption correction	Multi-scan (*SADABS*; Bruker, 2009 ▸)
*T*_min_, *T*_max_	0.640, 0.747
No. of measured, independent and observed [*I* > 2(*I*)] reflections	27415, 3667, 2884
*R*_int_	0.043
(sin /)_max_ (^1^)	0.694

Refinement	
*R*[*F* ^2^ > 2(*F* ^2^)], *wR*(*F* ^2^), *S*	0.035, 0.088, 1.03
No. of reflections	3667
No. of parameters	187
H-atom treatment	H-atom parameters constrained
_max_, _min_ (e ^3^)	0.70, 0.26

Computer programs: *APEX2* and *SAINT* (Bruker, 2009 ▸), *SHELXS97* and *SHELXL97* (Sheldrick, 2008 ▸), *ORTEP-3 for Windows* and *WinGX* (Farrugia, 2012 ▸).

## supplementary crystallographic information

### Crystal data

**Table:**

[Co(N_3_)_2_(C_12_H_8_N_4_S)_2_]	*F*(000) = 634
*M**_r_* = 623.56	*D*_x_ = 1.587 Mg m^−^^3^
Monoclinic, *P*2_1_/*c*	Mo *K*α radiation, λ = 0.71073 Å
Hall symbol: -P 2ybc	Cell parameters from 3667 reflections
*a* = 7.8004 (3) Å	θ = 2.6–29.6°
*b* = 8.2439 (3) Å	µ = 0.86 mm^−^^1^
*c* = 20.3222 (8) Å	*T* = 296 K
β = 92.910 (2)°	Block, red
*V* = 1305.15 (9) Å^3^	0.39 × 0.31 × 0.18 mm
*Z* = 2	

### Data collection

**Table:**

Bruker APEXII CCD diffractometer	3667 independent reflections
Radiation source: fine-focus sealed tube	2884 reflections with *I* > 2σ(*I*)
Graphite monochromator	*R*_int_ = 0.043
φ and ω scans	θ_max_ = 29.6°, θ_min_ = 2.6°
Absorption correction: multi-scan (*SADABS*; Bruker, 2009)	*h* = −8→10
*T*_min_ = 0.640, *T*_max_ = 0.747	*k* = −11→11
27415 measured reflections	*l* = −28→28

### Refinement

**Table:**

Refinement on *F*^2^	Primary atom site location: structure-invariant direct methods
Least-squares matrix: full	Secondary atom site location: difference Fourier map
*R*[*F*^2^ > 2σ(*F*^2^)] = 0.035	Hydrogen site location: inferred from neighbouring sites
*wR*(*F*^2^) = 0.088	H-atom parameters constrained
*S* = 1.03	*w* = 1/[σ^2^(*F*_o_^2^) + (0.0381*P*)^2^ + 0.5683*P*] where *P* = (*F*_o_^2^ + 2*F*_c_^2^)/3
3667 reflections	(Δ/σ)_max_ < 0.001
187 parameters	Δρ_max_ = 0.70 e Å^−^^3^
0 restraints	Δρ_min_ = −0.26 e Å^−^^3^

### Special details

**Table:**

Geometry. All e.s.d.'s (except the e.s.d. in the dihedral angle between two l.s. planes) are estimated using the full covariance matrix. The cell e.s.d.'s are taken into account individually in the estimation of e.s.d.'s in distances, angles and torsion angles; correlations between e.s.d.'s in cell parameters are only used when they are defined by crystal symmetry. An approximate (isotropic) treatment of cell e.s.d.'s is used for estimating e.s.d.'s involving l.s. planes.
Refinement. Refinement of *F*^2^ against all reflections. The weighted *R*-factor *wR* and goodness of fit *S* are based on *F*^2^, conventional *R*-factors *R* are based on *F*, with *F* set to zero for negative *F*^2^. The threshold expression of *F*^2^ > σ(*F*^2^) is used only for calculating *R*-factors(gt) *etc*. and is not relevant to the choice of reflections for refinement. *R*-factors based on *F*^2^ are statistically about twice as large as those based on *F*, and *R*- factors based on all data will be even larger.

### Fractional atomic coordinates and isotropic or equivalent isotropic displacement parameters (Å^2^)

**Table:**

	*x*	*y*	*z*	*U*_iso_*/*U*_eq_	
C1	0.7907 (3)	0.4986 (3)	0.07266 (12)	0.0486 (5)	
H1	0.8731	0.5035	0.0412	0.058*	
C2	0.8166 (3)	0.5770 (3)	0.13200 (13)	0.0540 (6)	
H2	0.9169	0.6353	0.1416	0.065*	
C3	0.6910 (3)	0.5672 (3)	0.17662 (13)	0.0568 (6)	
H3	0.7097	0.6203	0.2167	0.068*	
N1	0.5445 (2)	0.4873 (2)	0.16646 (9)	0.0448 (4)	
C5	0.5212 (2)	0.4114 (2)	0.10851 (9)	0.0316 (4)	
C6	0.3585 (2)	0.3225 (2)	0.10077 (8)	0.0298 (4)	
C7	0.0891 (2)	0.1991 (2)	0.11550 (8)	0.0285 (3)	
C8	−0.0761 (2)	0.1314 (2)	0.13257 (8)	0.0276 (3)	
C9	−0.1510 (2)	0.1627 (2)	0.19143 (8)	0.0338 (4)	
H9	−0.0963	0.2283	0.2233	0.041*	
C10	−0.3087 (2)	0.0943 (2)	0.20186 (9)	0.0369 (4)	
H10	−0.3623	0.1133	0.2410	0.044*	
C11	−0.3856 (2)	−0.0022 (2)	0.15379 (10)	0.0382 (4)	
H11	−0.4920	−0.0493	0.1599	0.046*	
C12	−0.3022 (2)	−0.0285 (2)	0.09601 (9)	0.0348 (4)	
H12	−0.3547	−0.0941	0.0637	0.042*	
C4	0.6406 (2)	0.4123 (2)	0.06039 (10)	0.0383 (4)	
H4	0.6204	0.3565	0.0210	0.046*	
N2	0.30958 (18)	0.23430 (19)	0.05046 (7)	0.0314 (3)	
N3	0.15296 (18)	0.16405 (19)	0.05884 (7)	0.0307 (3)	
N4	−0.14945 (18)	0.03680 (18)	0.08497 (7)	0.0285 (3)	
N5	0.1294 (2)	−0.1959 (2)	0.04761 (8)	0.0425 (4)	
N6	0.1687 (2)	−0.1872 (2)	0.10472 (8)	0.0394 (4)	
N7	0.2046 (3)	−0.1805 (3)	0.16093 (9)	0.0650 (6)	
S1	0.21579 (6)	0.32700 (6)	0.16328 (2)	0.03604 (12)	
Co1	0.0000	0.0000	0.0000	0.02677 (10)	

### Atomic displacement parameters (Å^2^)

**Table:**

	*U*^11^	*U*^22^	*U*^33^	*U*^12^	*U*^13^	*U*^23^
C1	0.0316 (10)	0.0552 (13)	0.0600 (14)	−0.0009 (10)	0.0134 (9)	0.0183 (11)
C2	0.0366 (11)	0.0422 (12)	0.0829 (17)	−0.0157 (10)	0.0016 (11)	0.0003 (12)
C3	0.0475 (13)	0.0543 (14)	0.0687 (15)	−0.0162 (11)	0.0041 (11)	−0.0261 (12)
N1	0.0358 (9)	0.0484 (10)	0.0508 (10)	−0.0103 (8)	0.0086 (8)	−0.0184 (8)
C5	0.0270 (8)	0.0304 (9)	0.0371 (9)	−0.0022 (7)	0.0009 (7)	0.0008 (7)
C6	0.0269 (8)	0.0349 (9)	0.0278 (8)	−0.0020 (7)	0.0028 (7)	0.0002 (7)
C7	0.0292 (8)	0.0349 (9)	0.0213 (7)	−0.0025 (7)	0.0004 (6)	−0.0024 (6)
C8	0.0277 (8)	0.0330 (9)	0.0223 (7)	−0.0009 (7)	0.0028 (6)	0.0013 (6)
C9	0.0368 (9)	0.0432 (10)	0.0216 (8)	−0.0008 (8)	0.0040 (7)	−0.0026 (7)
C10	0.0371 (9)	0.0468 (11)	0.0280 (9)	0.0032 (9)	0.0118 (7)	0.0033 (8)
C11	0.0308 (9)	0.0445 (11)	0.0403 (10)	−0.0032 (8)	0.0112 (8)	0.0055 (8)
C12	0.0310 (9)	0.0393 (10)	0.0342 (9)	−0.0060 (8)	0.0042 (7)	−0.0026 (7)
C4	0.0353 (9)	0.0443 (11)	0.0355 (10)	0.0012 (8)	0.0029 (8)	0.0038 (8)
N2	0.0284 (7)	0.0407 (8)	0.0252 (7)	−0.0078 (6)	0.0032 (6)	−0.0028 (6)
N3	0.0281 (7)	0.0411 (8)	0.0229 (7)	−0.0064 (6)	0.0028 (6)	−0.0027 (6)
N4	0.0283 (7)	0.0340 (7)	0.0235 (7)	−0.0032 (6)	0.0034 (6)	−0.0012 (6)
N5	0.0489 (10)	0.0472 (10)	0.0316 (8)	0.0043 (8)	0.0037 (7)	0.0004 (7)
N6	0.0301 (8)	0.0476 (10)	0.0404 (9)	−0.0064 (7)	0.0010 (7)	0.0125 (7)
N7	0.0610 (13)	0.0935 (17)	0.0391 (10)	−0.0169 (12)	−0.0125 (9)	0.0198 (10)
S1	0.0322 (2)	0.0481 (3)	0.0282 (2)	−0.0092 (2)	0.00487 (17)	−0.01202 (19)
Co1	0.02599 (16)	0.03633 (19)	0.01818 (15)	−0.00561 (14)	0.00290 (11)	−0.00329 (13)

### Geometric parameters (Å, º)

**Table:**

C1—C2	1.374 (3)	C9—H9	0.9300
C1—C4	1.382 (3)	C10—C11	1.374 (3)
C1—H1	0.9300	C10—H10	0.9300
C2—C3	1.371 (3)	C11—C12	1.388 (3)
C2—H2	0.9300	C11—H11	0.9300
C3—N1	1.326 (3)	C12—N4	1.337 (2)
C3—H3	0.9300	C12—H12	0.9300
N1—C5	1.338 (2)	C4—H4	0.9300
C5—C4	1.384 (3)	N2—N3	1.3704 (19)
C5—C6	1.467 (2)	N3—Co1	2.1301 (14)
C6—N2	1.297 (2)	N4—Co1	2.1535 (14)
C6—S1	1.7317 (16)	N5—N6	1.187 (2)
C7—N3	1.310 (2)	N5—Co1	2.1132 (17)
C7—C8	1.462 (2)	N6—N7	1.164 (2)
C7—S1	1.7128 (17)	Co1—N5^i^	2.1132 (17)
C8—N4	1.347 (2)	Co1—N3^i^	2.1301 (14)
C8—C9	1.382 (2)	Co1—N4^i^	2.1535 (14)
C9—C10	1.380 (3)		
			
C2—C1—C4	119.10 (19)	N4—C12—C11	122.64 (17)
C2—C1—H1	120.5	N4—C12—H12	118.7
C4—C1—H1	120.5	C11—C12—H12	118.7
C3—C2—C1	118.31 (19)	C1—C4—C5	118.00 (19)
C3—C2—H2	120.8	C1—C4—H4	121.0
C1—C2—H2	120.8	C5—C4—H4	121.0
N1—C3—C2	124.4 (2)	C6—N2—N3	111.59 (13)
N1—C3—H3	117.8	C7—N3—N2	113.40 (14)
C2—C3—H3	117.8	C7—N3—Co1	113.97 (11)
C3—N1—C5	116.54 (18)	N2—N3—Co1	132.53 (10)
N1—C5—C4	123.60 (17)	C12—N4—C8	117.54 (15)
N1—C5—C6	113.95 (15)	C12—N4—Co1	126.96 (12)
C4—C5—C6	122.44 (17)	C8—N4—Co1	115.44 (11)
N2—C6—C5	125.69 (15)	N6—N5—Co1	119.66 (14)
N2—C6—S1	114.57 (12)	N7—N6—N5	178.7 (2)
C5—C6—S1	119.72 (13)	C7—S1—C6	86.85 (8)
N3—C7—C8	120.22 (15)	N5—Co1—N5^i^	180.0
N3—C7—S1	113.57 (13)	N5—Co1—N3^i^	90.73 (6)
C8—C7—S1	126.20 (12)	N5^i^—Co1—N3^i^	89.27 (6)
N4—C8—C9	123.13 (16)	N5—Co1—N3	89.27 (6)
N4—C8—C7	113.46 (14)	N5^i^—Co1—N3	90.73 (6)
C9—C8—C7	123.40 (16)	N3^i^—Co1—N3	180.0
C10—C9—C8	118.44 (17)	N5—Co1—N4	90.35 (6)
C10—C9—H9	120.8	N5^i^—Co1—N4	89.65 (6)
C8—C9—H9	120.8	N3^i^—Co1—N4	103.24 (5)
C11—C10—C9	119.22 (16)	N3—Co1—N4	76.76 (5)
C11—C10—H10	120.4	N5—Co1—N4^i^	89.65 (6)
C9—C10—H10	120.4	N5^i^—Co1—N4^i^	90.34 (6)
C10—C11—C12	119.01 (17)	N3^i^—Co1—N4^i^	76.76 (5)
C10—C11—H11	120.5	N3—Co1—N4^i^	103.24 (5)
C12—C11—H11	120.5	N4—Co1—N4^i^	180.0

Symmetry code: (i) −*x*, −*y*, −*z*.

### Hydrogen-bond geometry (Å, º)

**Table:**

*D*—H···*A*	*D*—H	H···*A*	*D*···*A*	*D*—H···*A*
C2—H2···N6^ii^	0.93	2.59	3.432 (3)	151
C11—H11···N7^iii^	0.93	2.60	3.528 (3)	173
C10—H10···N1^iv^	0.93	2.63	3.438 (2)	146

Symmetry codes: (ii) *x*+1, *y*+1, *z*; (iii) *x*−1, *y*, *z*; (iv) −*x*, *y*−1/2, −*z*+1/2.
